# Emotional Intelligence of Undergraduate Athletes: The Role of Sports Experience

**DOI:** 10.3389/fpsyg.2021.609154

**Published:** 2021-01-28

**Authors:** Gabriel Rodriguez-Romo, Cecilia Blanco-Garcia, Ignacio Diez-Vega, Jorge Acebes-Sánchez

**Affiliations:** ^1^Faculty of Physical Activity and Sport Sciences (INEF), Universidad Politécnica de Madrid, Madrid, Spain; ^2^Departamento de Enfermería y Fisioterapia, Facultad de Ciencias de la Salud, Universidad de León, León, Spain; ^3^Faculty of Health Sciences, Universidad Francisco de Vitoria (UFV), Pozuelo de Alarcón, Spain

**Keywords:** emotional intelligence, sports experience, undergraduate athletes, TMMS-24, sports

## Abstract

Sport is an emotional experience. Studies have shown that high emotional intelligence (EI) is associated with better sports performance, though different aspects of sports experience and their relationship with EI are still unclear. This study examined the possible relationships between sports experience and EI dimensions of undergraduate athletes. Likewise, according to the differences described in the literature between men and women, the secondary aim was to identify the possible relationship between EI and sports experience in both subgroups. A total of 1784 [712 men (39.9%), 1072 women (60.1%); mean age = 21.3 years, SD = 4.2)] undergraduate athletes completed the Trait Meta Mood Scale and a sports experience questionnaire. Comparisons between groups were performed using Mann–Whitney-*U* and H-Kruskal–Wallis tests and correlations between variables were analyzed using Spearman correlation. We found that the number of different sports practiced and the number of years practicing sports were positively associated with emotional repair (ER). However, the number of years practicing sports was negatively associated with emotional attention (EA). Male athletes who trained more and had a higher competitive level were more likely to show higher ER. In any case, it is necessary to take into account that all the associations were weak. Our study suggested that athletes tend to attend to and value their feelings and use positive thinking to repair their negative moods.

## Introduction

In recent years, researchers have shown a growing interest in emotional intelligence (EI) and its relation to sports (Laborde et al., 2016a; Ubago-Jiménez et al., 2019). The concept of EI has been studied as a trait, an ability (Petrides, 2011) and as a mixed model (Mayer et al., 2008). Trait EI (trait emotional self-efficacy) refers to emotional self-perception and is measured using self-reporting questionnaires. Ability EI (cognitive-emotional ability) refers to cognitive abilities and is assessed through maximum performance testing (Petrides, 2011). The mixed model considers EI as a broad concept, consisting of motivation, intrapersonal and interpersonal abilities, empathy, personality factors, and wellness. The mixed model is assessed using self-reporting questionnaires (Mayer et al., 2008).

Laborde et al. (2016a), in their systematic review of sports, physical activity, and EI, found that the majority of studies (33 of 36) used self-report tools to assess EI. In other words, in the sport context, EI has been studied as a trait or as a mixed model on most occasions. This may be due to the dependence of ability EI tests on inherently subjective emotional experience (Matthews et al., 2007). Trait EI has been defined as a constellation of emotional self-perceptions at the lower levels of personality hierarchies (Petrides et al., 2007). Similarly, Mayer and Salovey (1997) proposed that EI consists of three dimensions: emotional attention (EA), emotional clarity (EC), and emotional repair (ER).

Laborde et al. (2013) described an emotion within the context of sports as an organized, psychophysiological reaction evaluating ongoing contextual relationships. Sports experiences are inherently emotional. Winning and losing, surpassing yourself through performance or recovering from an injury, all give rise to different feelings. Emotions are inherent to competition and can significantly influence performance (Hanin, 2000, 2010; Lazarus, 2000; Jones, 2003; Campo et al., 2012; Arribas-Galarraga et al., 2017; Magrum et al., 2019). Thus, EI may be a predictor of sports performance (Crombie et al., 2009; Kopp and Jekauc, 2018), and various techniques have been developed in the field of sports psychology to determine the optimal level of EI among athletes (Robazza et al., 2004; Gould and Maynard, 2009; Lane et al., 2009, 2010). The practice of sports puts emotions into play and may be a possible mechanism for the development of EI (Campo et al., 2016) given that every athlete reacts differently to these experiences.

There have been a number of studies into the difference in EI among athletes and non-athletes (Costarelli and Stamou, 2009; Szabo and Urban, 2014; Lepir et al., 2018). These studies found that athletes have greater EI than non-athletes, specifically in terms of assertiveness, understanding their own emotions, appraising others, and controlling their emotions.

Research carried out specifically with athletes has studied the relationships between EI and other variables related to sports experience, finding positive relationships between EI and higher athletic prowess (Saies et al., 2014; Arribas-Galarraga et al., 2017; Vaughan et al., 2019). Studies have also analyzed differences according to the type of sports (individual vs. team sports) and their relationship to EI (Kajbafnezhad et al., 2011; Ghaderi and Ghasemi, 2012; Laborde et al., 2014, 2017). While these studies found no significant differences according to the type of sports (individual vs. team sports), another study (Castro-Sánchez et al., 2018a) found significant differences in emotional management depending on the type of sports. Athletes practicing team contact sports (i.e., football, basketball, or rugby) showed higher levels of emotional management compared to athletes practicing individual contact sports (i.e., martial arts, combat sports). Castro-Sánchez et al. (2018b) found that the levels of EI are higher in team sports than in individual sports. This may be due to the differing psychological requirements of individual and team sports (Laborde et al., 2016b).

Other types of sports may have important nuances (Durand, 1975). Outdoor sports under extreme conditions can induce intense emotions (Johnson et al., 2016). Levels of EI have been studied in mountain/rock climbers (Ardahan, 2012; Marczak et al., 2017), mountain ultramarathon athletes (Nicolas et al., 2019), cyclists, and hikers (Ardahan, 2012), showing the importance of EI in outdoor sports performance. Studies of the association between EI and combat sports (Costarelli and Stamou, 2009; Szabo and Urban, 2014; Hyung et al., 2017; Merino et al., 2019, 2020) suggest that combat sports may also foster EI.

With regards to gender, sports and EI, various studies found no differences between male and female athletes (Laborde et al., 2014; Szabo and Urban, 2014; Castro-Sánchez et al., 2018a), neither in terms of their competitive level nor the type of sport (Laborde et al., 2016b, 2017). However, studies by Merino et al. (2019, 2020) found differing levels of EI among male and female athletes according to their competitive level; higher-level male athletes showed higher ER than the lower levels, but higher-level females athletes demonstrated increased EA and EC. It should be noted that higher EA scores are associated with excessive reactions to negative emotions (Yiend, 2009) and poorer emotional adjustment (Fernández-Berrocal et al., 2005). Another study with canoeists reported that men scored higher than women in emotional control and regulation (Arribas-Galarraga et al., 2017). Some studies suggest further lines of research in EI in the field of sports according to gender (Saies et al., 2014; Kopp and Jekauc, 2018).

Despite the existing research, various aspects of the relationship between sports experience and EI remain unclear (Costarelli and Stamou, 2009; Kajbafnezhad et al., 2011; Ghaderi and Ghasemi, 2012; Laborde et al., 2014, 2017; Saies et al., 2014; Szabo and Urban, 2014; Castro-Sánchez et al., 2018a,b; Lepir et al., 2018; Vaughan et al., 2019). The type of sport, time spent practicing (frequency days/week, years of practice), the number of different sports practiced, and the highest competitive level achieved are all characteristics of sports experience that may be related to EI. In contrast, some studies showed different results of EI within gender in the sport context (Laborde et al., 2014; Szabo and Urban, 2014; Castro-Sánchez et al., 2018a; Merino et al., 2019, Merino et al., 2020). Thus, this research aimed to analyze the association between different variables related to sports experience [type of sport (individual, team, outdoor, or combat), the number of years practicing sports, the training frequency, the number of different practiced sports and the level of competition achieved (not competitive, local/regional, or national/international)] and EI dimensions (EA, EC, and ER) of undergraduate athletes. The secondary aim was to describe the possible relationship between EI and sports experience in male and female athletes. Thus, the initial hypothesis is that there is a relationship between the sports experience and the EI of undergraduate athletes.

## Materials and Methods

### Sample and Procedure

A descriptive and cross-sectional study was conducted to analyze the associations between EI and sports experience. The population of the study was university students from Madrid. Disproportionate stratified sampling was used according to the type of university [public (79.1%) or private (20.9%)] and the subject areas of the students [social and legal sciences (41.2%), engineering and architecture (20.6%), arts and humanities (8%), and health sciences and science (25.8%)]. The sample consisted of 1784 [712 men (39.9%), 1072 women (60.1%); mean age = 21.3 years, SD = 4.2] undergraduate students from Madrid who claimed to be currently practicing at least one sport. Additionally, 58.7% reported practicing individual sports and 23.9% team sports; 55.3% of the students reported they trained more than 2 days/week while 39.5% reported training 1–2 days/week. The sample distribution data are presented in Table 1.

**TABLE 1:** Sample distribution data.

Variables	Total	Male	Female
**Type of university**			
*Public*	1411 (79.1)	565 (79.4)	846 (78.9)
*Private*	373 (20.9)	147 (20.6)	226 (21.2)
**Year course of studies^b^**			
*First*	459 (25.7)	196 (27.5)	263 (24.5)
*Second*	413 (23.2)	177 (24.9)	236 (22)
*Third*	384 (21.5)	144 (20.2)	240 (22.4)
*Fourth*	404 (22.6)	152 (21.3)	252 (23.5)
*Fifth*	83 (4.7)	31 (4.4)	52 (4.9)
*Sixth*	41 (2.3)	12 (1.7)	29 (2.7)
**Subject area**			
*Arts and humanities*	143 (8)	30 (4.2)	113 (10.5)
*Sciences*	77 (4.3)	37 (5.2)	40 (3.7)
*Health sciences*	461 (25.8)	175 (24.6)	286 (26.7)
*Social and legal sciences*	735 (41.2)	257 (36.1)	478 (44.6)
*Engineering and architecture*	368 (20.6)	213 (29.9)	155 (14.5)
Number of practiced sports^a^	1 (1–2)	2 (1–2)	1 (1–2)
Years practicing sports^a^	16 (8–18)	14 (7–18)	16 (10–19)
Starting age of sports practice^a^	4 (2–10)	6 (3–12.5)	3.75 (1.75–8)
**Training frequency^b^**			
*Seasonal*	41 (2.3)	13 (1.8)	28 (2.6)
*Less than 1 day/week*	51 (2.9)	22 (3.1)	29 (2.7)
*1*–*2 days/week*	705 (39.5)	231 (32.4)	474 (44.2)
*More than 2 days/week*	987 (55.3)	446 (62.6)	541 (50.5)
**Type of sport^b^**			
*Individual*	1048 (58.7)	301 (42.3)	747 (69.7)
*Team*	427 (23.9)	286 (40.2)	141 (13.2)
*Combat*	131 (7.3)	69 (9.7)	62 (5.8)
*Outdoor*	96 (5.4)	34 (4.8)	62 (5.8)
*Others*	82 (4.6)	22 (3.1)	60 (5.6)
**Competitive level^b^**			
*Not competitive*	1109 (62.2)	327 (45.9)	782 (72.9)
*Local/regional*	469 (26.3)	279 (39.2)	190 (17.7)
*National/international*	206 (11.6)	106 (14.9)	100 (9.3)

*Results are expressed in: ^*a*^median (Quartile 1–Quartile 3); ^*b*^frequency (percentage).*

Participation was voluntary and confidential, and informed consent was obtained from participants before completing the survey. This study was approved by the Research Ethics Committee of Universidad Francisco de Vitoria (40/2018).

Participants were recruited by their lecturers, who sent them a Google Forms Questionnaire. Participants completed the sports experience survey and EI questionnaire. The sample was collected from April to December 2017 at Madrid universities.

### Measures

#### Emotional Intelligence

Undergraduate students were assessed using the validated Spanish version of the Trait Meta-Mood Scale (TMMS-24) (Fernández-Berrocal et al., 2004) based on the original scale developed by Salovey et al. (1995). This self-reporting tool contains 24 items using a 5-point Likert scale from 1 (totally disagree) to 5 (totally agree). It is composed of three dimensions (eight items each dimension): (1) emotional attention (EA), evaluating how people attend to and value their feelings (item example: “I pay a lot of attention to my feelings”); (2) emotional clarity (EC), evaluating how people feel clearly rather than confusedly about their feelings (item example: “I can often define my feelings”), and (3) emotional repair (ER), evaluating how people use positive thinking to repair negative moods (item example: “Although sometimes I feel sad, I usually have an optimistic vision”). Our results demonstrate similar internal consistency in the three sub-scales: EA, α = 0.89; EC, α = 0.90; and ER, α = 0.83.

#### Sports Experience

The variables for sports experience were the number of different sports practiced, the number of years practicing sports, the training frequency, the type of sport practiced, and the maximum level of competition achieved in this sport. We assessed the training frequency by self-reporting on a Likert scale from 1 (less than 1 day/week) to 3 (more than 2 days/week); there was a 4th for seasonal sports. To evaluate the type of sport, the participants had to choose a sport from a list of 43 sports; the selected sports were then categorized into individual, team, combat, and outdoor sports following the classification by Durand (1975). The level of competition was assessed by self-reporting using three categories: 1 (not competitive), 2 (local and regional level), and 3 (national and international level). The questionnaire also asked for the number of different sports practiced and the number of years practicing sports.

### Data Analysis

The data is presented as a median and interquartile range. EI variables showed a non-normal distribution. Comparisons between groups were made using Mann–Whitney-U and H-Kruskal–Wallis tests. When necessary we performed Dunn-Bonferroni *post hoc* tests. Correlations between the variables were analyzed using Spearman correlation.

The data were analyzed using the Statistical Package for the Social Sciences (SPSS v21). Statistical significance was set at *p* < 0.05. However, as EI was consists of three dimensions, the significance level was adjusted at *p* < 0.016. D-Cohen was used to interpret the effect size, defining small (*d* = 0.2), medium (*d* = 0.5), and large (*d* = 0.8) values.

## Results

For the EI dimensions median values were EA = 29 (25–33), EC = 28 (24–32), and ER = 29 (25–32). Table 2 shows the associations between sports experience and EI dimensions. There was a significant positive association between the number of sports practiced and the ER (*p* < 0.001). There was a significant negative association between the number of years practicing sports and EA (*p* = 0.002) and a significant positive association with ER (*p* = 0.012). The analysis showed significant differences among training frequency groups and ER (*p* < 0.001), but there are no post-hoc differences between groups that were statistically identified. There were significant differences between the type of sports and EA (*p* < 0.001). Individual sports showed higher EA than team sports (*p* < 0.001). There were significant differences between the competitive level and EA (*p* < 0.001). Participants who did not compete showed higher EA than participants who compete at local/regional level (*p* < 0.001) and participants who compete at national/international level (*p* = 0.011).

**TABLE 2:** Associations and differences between emotional intelligence dimensions and sports experience.

	EA	EC	ER	p			d		
				EA	EC	ER	EA	EC	ER
**Number of practiced sports^a^**	−0.038	0.035	0.076	0.11	0.142	**0.001**	0.08	0.07	0.15
**Years practicing sports^a^**	−0.073	0.021	0.059	**0.002**	0.382	**0.012**	0.15	0.04	0.12
**Starting age of sports practice^a^**	0.067	0.042	0.008	**0.004**	0.077	0.74	0.13	0.08	0.02
**Training frequency**									
*Seasonal*^1^	30 (25–35)	28 (23–31)	28 (23–30)	0.092	0.046	**<0.001**	0.09	0.11	0.18
*Less than 1 day/week^2^*	29 (23–32)	28 (22–32)	29 (22.5–32)						
*1*–*2 days/week^3^*	30 (25–33)	28 (23–32)	28 (24–32)						
*More than 2 days/week^4^*	29 (24–33)	28 (24–32)	29 (25–33)						
**Type of sport**									
*Individual*^1^	30 (25–34)^2^	28 (24–32)	29 (25–33)	**<0.001**	0.865	0.069	0.23	0.08	0.10
*Team*^2^	28 (24–32)^1^	28 (24–32)	28 (24–32)						
*Combat*^3^	29 (25–33)	28 (23–32)	29 (24–32)						
*Outdoor*^4^	29 (24.5–33)	28 (24–32)	29 (24.5–32)						
*Others*^5^	29.5 (25–34)	28 (23–32)	27 (23–31)						
**Competitive level**									
*Not competitive^1^*	30 (25–34)^2,3^	28 (24–32)	29 (25–32)	**<0.001**	0.207	0.028	0.21	0.05	0.11
*Local/regional^2^*	28 (24–32)^1^	28 (23–32)	28 (24–32)						
*National/international^3^*	29 (25–32)^1^	28 (24–32)	30 (26–33)						

*Results are expressed in median (Quartile 1–Quartile 3). ^*a*^Results are Spearman correlation coefficients. EA, emotional attention; EC, emotional clarity; ER, emotional repair; p, *p*-value; d, *d*-Cohen. Superscript numbers reflect statistically significant differences between categories with Bonferroni adjustment in each EI dimensions. Bold values are statistically significant results (*p* < 0.016).*

The median EI score for male athletes was 28 (9) in EA, 29 (8) in EC, and 29 (8) in ER. Among female athletes, scores were 30 (8) in EA, 28 (9) in EC, and 28 (8) in ER. Significant differences were found between male and female athletes in the three dimensions of emotional intelligence (*p* < 0.001). Female athletes scored higher in EA while male athletes were higher in EC and ER. Tables 3, 4 show the relationships between the profile of sports practice and the dimensions of EI according to gender.

**TABLE 3:** Associations and differences between emotional intelligence dimensions and sports experience for male athletes.

	EA	EC	ER	p		d			
				EA	EC	ER	EA	EC	ER
**Number of practiced sports^a^**	−0.001	0.008	0.065	0.98	0.823	0.085	<0.01	0.02	0.13
**Years practicing sports^a^**	−0.089	0.017	0.039	0.018	0.646	0.299	0.18	0.03	0.08
**Starting age of sports practice^a^**	0.079	0.01	−0.015	0.036	0.8	0.691	0.16	0.02	0.03
**Training frequency**									
*Seasonal*^1^	31 (30–36)	29 (24–33)	28 (23–30)						
*Less than 1 day/week^2^*	27.5 (22–32)	30 (24–32)	30.5 (24–32)	0.157	0.358	**0.002**	0.11	0.04	0.26
*1*–*2 days/week^3^*	28 (24–32)	28 (24–32)	28 (24–32)^4^						
*More than 2 days/week^4^*	27 (23–32)	29 (25–32)	30 (26–33)^3^						
**Type of sport**									
*Individual*^1^	28 (24–33)^2^	30 (25–32)	30 (25–33)	**0.010**	0.316	0.257	0.23	0.07	0.09
*Team*^2^	27 (22–31)^1^	28 (24–32)	28.5 (25–32)						
*Combat*^3^	30 (25–33)	29 (23–32)	29 (24–32)						
*Outdoor*^4^	26 (24–32)	30 (25–32)	29 (26–32)						
*Others*^5^	28.5 (25–31)	28 (24–31)	29.5 (26–34)						
**Competitive level**									
*Not competitive^1^*	28 (24–33)	29 (25–32)	29 (25–33)	0.162	0.375	**0.013**	0.10	0.02	0.20
*Local/regional^2^*	27 (22.5–32)	28 (24–32)	28 (24–32)^3^						
*National/international^3^*	28 (24–31)	29 (26–32)	30 (27–33)^2^						

*Results are expressed in median (Quartile 1–Quartile 3). ^*a*^Results are Spearman correlation coefficients. EA, emotional attention; EC, emotional clarity; ER, emotional repair; p, *p*-value; d, *d*-Cohen. Superscript numbers reflect statistically significant differences between categories with Bonferroni adjustment in each EI dimensions. Bold values are statistically significant results (*p* < 0.016).*

**TABLE 4:** Associations and differences between emotional intelligence dimensions and sports experience for female athletes.

	EA	EC	ER	p			d		
				EA	EC	ER	EA	EC	ER
**Number of practiced sports^*a*^**	−0.026	0.028	0.066	0.395	0.356	0.030	0.05	0.06	0.13
**Years practicing sports^*a*^**	−0.009	−0.01	0.058	0.757	0.746	0.059	0.02	0.02	0.12
**Starting age of sports practice^*a*^**	0.023	0.094	0.046	0.458	**0.002**	0.128	0.05	0.19	0.09
**Training frequency**									
*Seasonal*^1^	29.5 (25–33.5)	27 (22–31)	27.5 (23.5–31.5)						
*Less than 1 day/week^2^*	30 (25–36)	27 (22–32)	28 (22–30)	0.376	0.321	0.103	0.02	0.04	0.11
*1*–*2 days/week^3^*	30 (26–34)	27 (23–32)	28 (24–32)						
*More than 2 days/week^4^*	30 (25–34)	28 (24–32)	29 (25–32)						
**Type of sport**									
*Individual*^1^	30 (26–34)	28 (23–32)	29 (25–32)	0.107	0.463	0.044	0.12	0.04	0.15
*Team*^2^	30 (26–33)	27 (23–30)	28 (24–32)						
*Combat*^3^	29 (23–31)	27.5 (23–32)	28 (24–32)						
*Outdoor*^4^	30 (25–34)	27 (24–31)	28 (23–32)						
*Others*^5^	30.5 (25.5–34)	27.5 (22.5–32)	27 (22.5–30)						
**Competitive level**									
*Not competitive^1^*	30 (26–34)	28 (23–32)	29 (25–32)	0.064	0.056	0.579	0.11	0.12	0.06
*Local/regional^2^*	29 (25–33)	27 (22–31)	28 (24–32)						
*National/international^3^*	29 (26–33)	27 (24–31.5)	29 (25–34)						

*Results are expressed in median (Quartile 1–Quartile 3). ^*a*^Results are Spearman correlation coefficients. EA, emotional attention; EC, emotional clarity; ER, emotional repair; p, *p*-value; d, *d*-Cohen. Superscript numbers reflect statistically significant differences between categories with Bonferroni adjustment in each EI dimensions. Bold values are statistically significant results (*p* < 0.016).*

For the male athlete group, our results showed relationships between ER and training frequency (*p* = 0.002) and the competitive level (*p* = 0.013); relationships were also found between EA and the type of sport (*p* = 0.01). Male athletes who do sports for more than 2 days/week had higher ER than those who do sport 1 or 2 days a week. Those that compete at a national or international level had better ER than those that compete at the local or regional level; male athletes who practice an individual sport had higher EA than male athletes who practice team sports (Table 3).

For female athletes, there was a relationship between EC and the age of starting practicing sports (*p* = 0.002). Thus, female athletes who started sports later had higher outcomes in EC (Table 4).

## Discussion

This study analyzed the possible associations between sports experience (the type of sport; the number of years participating in sports; the number of different sports practiced; the highest level achieved in competition) and dimensions of EI (EA, EC, and ER) among undergraduate athletes.

The findings suggest significantly higher EA among athletes practicing individual sports compared to team sports. However, this relationship was weak. Previous studies showed no significant differences between the team and individual sports (Kajbafnezhad et al., 2011; Ghaderi and Ghasemi, 2012; Laborde et al., 2014, 2017). Likewise, Castro-Sánchez et al. (2018a; 2018b) found that EI correlates more strongly with team sports than individual sports athletes, finding significant differences in emotional self-management (similar to ER, assessed using the Schutte Self Report Inventory). However, there was no significant difference in emotional perception (similar to EA, assessed using the Schutte Self Report Inventory). Our results suggest that athletes practicing individual sports tend to observe and think about their feelings and moods more than athletes of team sports. Those athletes who face the emotional demands of sports autonomously probably feel and express their emotions more easily than those who are subjected to the judgment of teams or leaders. This may be a problem since higher EA is related to excessive reactions to negative emotions (Yiend, 2009) and poorer emotional adjustment (Fernández-Berrocal et al., 2005). Athletes practicing individual sports face their sporting experiences alone and, thus, some individual athletes tend to ruminate on their mistakes and criticized themselves, creating a loop of negative emotions. However, when team sport athletes make mistakes, the group can help their teammates into a better emotional state. This may be interesting for sports psychologist interventions depending on the type of sport. Furthermore, our results also suggest that the experience, measured in quantity (number of years practicing sports) and the quality of the experience (competition level), is related to a lower EA. Thus, experience and sporting prowess may be associated with better emotional adjustment (Fernández-Berrocal et al., 2005; Laborde et al., 2011). This may be because the demands of the sport lead the athlete to maintain an optimal emotional state for his performance. These results are in line with the findings showing that experts cope better with stress (Johnson et al., 2006; Laborde et al., 2013).

Our results showed that the number of years practicing sports is significantly and positively correlated to a higher ER, although the size effect was weak, similar to the majority of the associations found in our research. Laborde et al. (2014) found no correlation between total EI and the number of years of sports training. However, assuming that high-level athletes practice more than others, various studies have found that high-level athletes show higher ER than low-level athletes (Saies et al., 2014; Merino et al., 2019, 2020). This may be because strategies to regulate emotions become crucial in sports (Jones, 2003), and a number of techniques have been developed in sports psychology aimed to achieve optimal performance (Lane et al., 2010). These techniques may be learned by athletes to improve their performance pre, during, and post-competition (Robazza et al., 2004; Gould and Maynard, 2009). As seen in other articles (Saies et al., 2014; Merino et al., 2019), one could assume that a higher level of competition is associated with higher ER; however, our results have not been significant. This data is consistent because athletes who practice more years and different sports showed higher levels of ER. Furthermore, there is an association between ER and the ability to control intrusive and ruminative thoughts that, often, accompany stressful situations (Salovey et al., 1995). This could be crucial for sporting performance, as those athletes who recover from negative emotional states will perform better.

Regarding EC, no associations or relations were found with any studied variable. We should highlight that the results are within the reference values for all EI dimensions. These parameters were described by the TMMS-24 tool (Fernández-Berrocal et al., 2004). However, ER and EC could be improved to reach excellent values. It seems that, from a performance point of view, it is more interesting for athletes to attend to and value their feelings and use positive thoughts to repair negative moods, instead to feel their feelings clearly.

Due to the controversial results between sports experience and EI variables, more studies should be carried out to understand these relationships more deeply which could be of great use to sports psychologist and coaches even though previous studies have demonstrated that EI training can be improved in the sports context (Lane et al., 2009; Campo et al., 2016).

The secondary aim of the present study was to describe the possible relationship between EI and sports experience in male and female athletes. The differences found between levels of EI is supported by previous studies (Laborde et al., 2014, 2016b, 2017; Castro-Sánchez et al., 2018a,b), and they encourage further study into the possible relationships between EI and sports experience manifested themselves in the subgroups (male and female athletes). Female athletes achieved higher scores in EA, which is in line with the findings of Merino et al. (2020) in combat sports. However, this study found no differences in EC and ER domains where our results showed that male athletes had significantly higher EC and ER than female athletes. Male athletes who trained more and had a higher competitive level are more likely to show higher ER. Thus, ER may be related to a higher sports performance than the other dimensions (EA and EC) for male athletes. These results are partly in line with the findings of Merino et al. (2020) in which athletes from the high-level group showed higher ER and EC than low-level athletes. Our results support the idea that different strategies according to gender should be considered in the context of sports to improve performance related EI skills (Hanin, 2000, 2010; Lazarus, 2000; Jones, 2003; Campo et al., 2012).

Our study has some limitations. The cross-sectional design means that we were unable to infer causal relationships among the analyzed variables. Longitudinal studies would be required to establish cause-effect relationships and track the changes in EI during sports practice. However, a methodological strength of the study is the use of TMMS-24 as a measurement instrument in a large sample of undergraduates in Madrid, which was representative in terms of academic disciplines. The statistical analyses were limited because of the difference between the compared groups. The data did not meet the assumptions to carry out parametric tests. These facts compromised the study of interactions, which could have been carried out with some types of factorial MANOVA or linear or polynomial regressions. So, we recommend taking these limitations into account to improve the design of future research. Furthermore, it should be noted that the effect sizes were low in all significant associations. Future research should study how different sports experience variables are related to EI dimensions and how these relationships can be module according to gender within large samples and specifically among sports modalities. Future studies should identify which EI dimensions are related to high performance among sports modalities. This information would be useful for coaches and sports psychologists who work with high-performance athletes because, under highly equitable technical and tactical circumstances, adequate emotional management could make the difference between winning or losing (Magrum et al., 2019).

Despite its limitations, the current study provides insight into the potential relationships between sports experience and EI. Our research did not find a close relationship but did find certain degrees of associations. Specifically, those athletes who play individual sports for fewer years and who do not compete show a higher EA, while ER was positively related to the number of years practicing sports and the number of sports practiced. No associations were found between EC and sports experience. Regarding gender, EI scores showed significant differences. Female athletes showed higher EA and male athletes showed higher EC and ER. Furthermore, relationships between EI and sports experience in male athletes showed that men who trained more and had a higher competitive level were more likely to show higher ER. Therefore, it is likely that ER may be related to higher sports performance than the other EI dimensions (EA and EC) for male athletes. However, all of these relationships were found to be weak, and thus, the conclusions of the present study should be interpreted considering this premise. Consequently, more research is needed to understand how these relationships work.

## Data Availability Statement

The raw data supporting the conclusions of this article will be made available by the authors, without undue reservation.

## Ethics Statement

The studies involving human participants were reviewed and approved by the Ethics Research Committee of Universidad Francisco de Vitoria (40/2018). The patients/participants provided their written informed consent to participate in this study.

## Author Contributions

GR-R, CB-G, and JA-S: conceptualization. ID-V: formal analysis. GR-R: funding acquisition and project administration. JA-S: investigation and supervision. ID-V, GR-R, and JA-S: methodology and writing – original draft. CB-G, ID-V, JA-S, and GR-R: writing – review and editing. All authors contributed to the article and approved the submitted version.

## Conflict of Interest

The authors declare that the research was conducted in the absence of any commercial or financial relationships that could be construed as a potential conflict of interest.
